# Dysregulation of LINC00470 and METTL3 promotes chemoresistance and suppresses autophagy of chronic myelocytic leukaemia cells

**DOI:** 10.1111/jcmm.16478

**Published:** 2021-03-21

**Authors:** Xun Lai, Jia Wei, Xue‐zhong Gu, Xiang‐mei Yao, Di‐si Zhang, Feng Li, Yun‐yan Sun

**Affiliations:** ^1^ Department of Hematology The Third Affiliated Hospital of Kunming Medical University (Tumor Hospital of Yunnan Province) Kunming China; ^2^ Department of Hematology The First People’s Hospital of Yunnan Province Kunming China

**Keywords:** AKT, autophagy, chemoresistance, Chronic myelocytic leukaemia, LINC00470, METTL3, PTEN

## Abstract

Cytoplasmic lncRNAs have been found to directly interact with target mRNAs and regulate their stability. In this study, we aimed to study the molecular mechanism underlying the function of m^6^A as a central regulator in chemoresistance and CML proliferation. In this study, we established three mice groups (control group, ADR‐R group and ADR‐R + shLINC00470 group). We detected PTEN mRNA expression in the presence of LINC00470 in the mice models, as well as in the KCL22 and K562 cells. LINC00470 was significantly enriched for PTEN mRNA to exhibit a negative regulatory relationship between LINC00470 and PTEN mRNA. However, the alteration of LINC00470 had no effect on the luciferase activity of PTEN promoter, while the half‐life of PTEN mRNA was affected. It was further validated that LINC00470 down‐regulated PTEN expression by positively regulating the m6A modification of PTEN mRNA via RNA methyltransferase METTL3. Moreover, the relative expression of LC3II, Beclin‐1, ATG7 and ATG5 was all decreased in cells treated with LINC00470, and down‐regulated PTEN expression was observed in chemo‐resistant cells, while the expression of PTEN was rescued by the transfection of shMETTL3 into chemo‐resistant cells. Moreover, the knockdown of METTL3 also restored the normal level of PTEN m^6^A modification and LINC00470 expression in chemo‐resistant cells. In conclusion, our results demonstrated the molecular mechanism underlying the effect of LINC00470 on CML by reducing the PTEN stability via RNA methyltransferase METTL3, thus leading to the inhibition of cell autophagy while promoting chemoresistance in CML.

## 1 INTRODUCTION

As a type of bone marrow malignancy featured by the generation of breakpoint cluster region (BCR) as well as the formation of Abelson murine leukemia (ABL) gene encoding BCRABLp210, chronic myeloid leukaemia (CML) can be specifically divided into 3 phases, that is chronic phase (CP), accelerated period (AP), as well as blast phase (BP), while advanced CML is characterized by the AP and BP phases of CML.^1^, ^2^, ^3^

Long non‐coding RNAs, also termed lncRNAs, belong to a type of RNAs with no coding capacity. LncRNAs contain >200 nucleotides in their transcripts and may interact with target mRNAs, microRNAs, and proteins to control gene transcription, chromatin modification, as well as post‐transcriptional and post‐translational gene expression.^4^ The abnormality in lncRNA expression was found to be linked to many types of cancers, and hence may be used as the prognostic biomarker for cancer diagnosis.^5^, ^6^ LncRNA LINC00470 is located between RP11‐732L14 and RP11‐16P11 loci in chromosome 18p11.32.^7^, ^8^ The different splicing modes of the seven exons in lncRNA LINC00470 can generate four types of transcripts. It was previously shown that the expression of LINC00470 in astrocytoma was significantly elevated compared to that in typical brain tissues.^9^ LINC00470 is related to the mRNA of phosphatase and tensin homologue (PTEN) and can recruit the binding of m6A methyltransferase METTL3 to PTEN mRNA, thus increasing the level m6A methylation in PTEN mRNA while enhancing the interaction between PTEN mRNA and YTHDF2.^10^

Methyltransferase like 3 (METTL3) has been characterized as the primary methyltransferase vital for the process of m6A methylation.^11^ The knockdown or overexpression of METTL3 modifies the level of global m6A methylation, direct impacting the decay as well as translation in the biogenesis of microRNA and mRNA, and resulting in the development of human cancer.^11^ It was found that the inhibition of METTL3 expression reduced the level of m6A methylation in the genes of c‐MYC, PTEN and BCL2. Consistently, it was found that the depletion of METTL3 using shRNA specific to METTL3 resulted in significant reduction in the expression of cMYC, PTEN and BCL2 proteins.^12^

PTEN acts as a significant tumour suppressor in the human body.^13^, ^14^, ^15^ A canonical function of PTEN is to suppress the activation of the phosphatidylinositol 3‐kinase (PI3K)‐AKT signalling via the phosphatase activity of PTEN, subsequently inhibiting the activity of AKT as well as the signalling of its downstream pathways. Lately, PTEN‐Long (PTEN‐L), a long form of PTEN produced by translation from an alternate upstream start codon, was recognized as a new PTEN isoform.^16^, ^17^, ^18^ In addition, the results shown in a recent article presented that ATM controls the phosphorylation of PTEN induced by DNA‐damaging substances at the Ser113 site, and subsequently promotes the nuclear translocation of PTEN to promote autophagy of tumour cells via the activation of p‐JUN‐SESN2‐AMPK signalling.^19^ Given that PTEN‐Lis is evolutionarily conserved across many species, the polymorphic variants of PTEN or its mutations may exert a considerable effect on the function of PTENL.^20^ The canonical role of PTEN in CML patients also suggested that there was no direct link between the expression of PTEN gene and the resistance to chemotherapy.^21^ And PTEN has been reported to be closely associated with the BCR‐ABL‐induced leukaemias in the treatment of Philadelphia chromosome‐positive leukaemia due to its role as a tumour suppressor in CML.^22^ Nevertheless, reduced expression level of PTEN protein was observed in a CML mouse model.^23^ Given that the function of PTEN‐L is significant in leukaemogenesis, functional studies are needed to clarify the involvement of PTEN‐L in the pathogenesis of CLM.^24^, ^25^

METTL3 was reported to be involved in tumorigenesis of various malignancies including gastric cancer, liver cancer, hepatoblastoma, colorectal cancer and CML, and LINC00470 was found to be a regulator of METTL3.^10^, ^26^, ^27^, ^28^, ^29^ In this study, we used a CML cell line and an animal exoplant model of CML to study the effect of LINC00470 on the expression of METTL3 and its involvement in the pathogenesis of CML.

## 2 MATERIALS AND METHODS

### 2.1 Animal model

In this study, three groups of BALB/c male nude mice were established as the control mice group (transplanted with parental K562 cells), ADR‐R mice group (transplanted with chemo‐resistant K562 cells) and ADR‐R + shLINC00470 group (transplanted with chemo‐resistant cells that were transfected with shLINC00470), respectively. In the control mice or ADR mice group, the parental or chemo‐resistant K562 cells were infected with LV‐shCtrl. In the ADR + shLINC00470 group, the chemo‐resistant K562 cells were infected with LV‐ shLINC00470. These cells were injected, respectively, into these 5‐week‐old mice subcutaneously (1.5 × 10^7^ cells per mouse with 9 mice in each group). Meanwhile, three parallel animal groups were set up, in which the mice also received ADR treatment apart from the inoculation of K562 cells. Institutional ethical committee has approved the protocol of this study.

### 2.2 Cell culture and transfection

In this study, KCL22 and K562 cell lines were acquired from ATCC and cultured in a standard RPMI 1640 medium (Gibco, Thermo Fisher Scientific) supplemented with 100 μg/ml of streptomycin, 100 IU/ml of penicillin, and 10% of heat inactivated FBS. The cells were maintained at 37°C in a humidified environment containing 5% CO_2_ and 95% air. When the cultured KCL22 and K562 cells reached 70% confluency, they were seeded at a concentration of 1.5 × 10^6^ cells/mL in 6‐well plates, with 2 mL of cell suspension added into each well. To study the role of LINC00470 in regulating the expression of LC3II, Beclin‐1, ATG7 and ATG5 via RNA methyltransferase METTL3, several cell models were established as below. As it was shown that cytoplasmic lncRNAs could directly interact with target mRNAs to regulate their stability,^12^ an RIP assay was carried out in this study using an MS2‐binding protein (MS2bp) targeting RNAs carrying the MS2‐binding sequence (MS2bs). In brief, the MS2bs elements were inserted into a plasmid carrying the full‐length sequence of LINC00470, and the plasmid was co‐transfected into KCL22 and K562 cells along with the plasmid carrying the MS2bp‐GFP gene. In cell model I, KCL22 and K562 cells were divided into four groups, that is (1) MS2‐IgG group (KCL22 and K562 cells transfected with the MS2‐IgG plasmid); (2) MS2‐anti‐GFP group (KCL22 and K562 cells transfected with the MS2‐anti‐GFP plasmid); (3) LINC00470‐MS2‐IgG group (KCL22 and K562 cells transfected with the LINC00470‐MS2‐IgG plasmid); and (4) LINC00470‐MS2‐anti‐GFP group (KCL22 and K562 cells transfected with the LINC00470‐MS2‐anti‐GFP plasmid). In cell model II, a plasmid carrying the full‐length sequence of LINC00470 as well as the MS2bs elements was co‐transfected into KCL22 and K562 cells along with a plasmid carrying MS2bp‐GFP. The KCL22 and K562 cells were divided into two groups, that is (1) Beads group (KCL22 and K562 cells transfected with beads) and (2) LINC00470 group (KCL22 and K562 cells transfected with the plasmid carrying the full‐length sequence of LINC00470 as well as the MS2bs elements). In cell model III, the KCL22 and K562 cells were also divided into two groups, that is (1) shNC group (KCL22 and K562 cells transfected with a negative control shRNA shNC); and (2) shLINC00470 group (KCL22 and K562 cells transfected with the plasmid carrying shLINC00470). In cell model IV, the KCL22 and K562 cells were still divided into two groups, that is (1) NC group (KCL22 and K562 cells transfected with a negative control plasmid) and (2) LINC00470 group (KCL22 and K562 cells transfected with the plasmid carrying LINC00470). To study the relationship between LINC00470 and PTEN, a luciferase reporter assay was carried out using the luciferase reporter plasmid carrying the promoter of PTEN, which was co‐transfected into KCL22 and K562 cells with shLINC00470 or shNC. In cell model V, the KCL22 and K562 cells were divided into four groups, that is (1) shNC + pGL3 group (KCL22 and K562 cells co‐transfected with the negative control shRNA shNC and the empty luciferase reporter plasmid pGL3); (2) shLINC00470 + pGL3 group (KCL22 and K562 cells co‐transfected with shLINC00470 and the empty luciferase reporter plasmid pGL3); (3) shNC + pGL3‐PTEN group (KCL22 and K562 cells co‐transfected with the negative control shRNA shNC and the luciferase reporter plasmid pGL3‐PTEN); and (4) shLINC00470 + pGL3‐PTEN group (KCL22 and K562 cells co‐transfected with shLINC00470 and the luciferase reporter plasmid pGL3‐PTEN). In cell model VI, the KCL22 and K562 cells were divided into four groups, that is (1) NC + pGL3 group (KCL22 and K562 cells co‐transfected with the negative control and the empty luciferase reporter plasmid pGL3); (2) LINC00470 + pGL3 group (KCL22 and K562 cells co‐transfected with LINC00470 and the empty luciferase reporter plasmid pGL3); (3) NC + pGL3‐PTEN group (KCL22 and K562 cells co‐transfected with the negative control and the luciferase reporter plasmid pGL3‐PTEN); and (4). LINC00470 + pGL3‐PTEN group (KCL22 and K562 cells co‐transfected with LINC00470 and the luciferase reporter plasmid pGL3‐PTEN). In cell model VII, the KCL22 and K562 cells were divided into two groups, that is (1) IgG group (KCL22 and K562 cells treated with anti‐IgG antibody); and (2) M6A group (KCL22 and K562 cells treated with anti‐M6A antibody. In cell model VIII, the KCL22 and K562 cells were divided into four groups, that is (1) IgG group (KCL22 and K562 cells treated with anti‐IgG antibody); (2) METTL3 group (KCL22 and K562 cells treated with anti‐METTL3 antibody); (3) METTL14 group (KCL22 and K562 cells treated with anti‐METTL14 antibody); and (4) WTAP group (KCL22 and K562 cells treated with anti‐WTAP antibody). All cell transfections in this study were carried out by using the Lipofectamine RNAiMAX transfection reagent (Invitrogen, Carlsbad, CA) based on the directions provided by the transfection reagent manufacturer on the product manual. After 48 hours of transfection, the transfected cells were collected for the analysis of target genes.

### 2.3 RNA isolation and real‐time PCR

In this study, real‐time PCR was carried out to assay the expression of PTEN mRNA in each sample. In brief, total RNA was extracted using a Trizol reagent (Invitrogen) based on the directions provided by the reagent manufacturer on the product manual without employing other purification and enrichment of isolated RNA. Then, using the AMV reverse transcriptase purchased from New England Biolabs (Ipswich, MA), isolated RNA was converted into cDNA based on the directions provided by the manufacturer of reverse transcriptase. Using the cDNA as the template, real‐time PCR was carried out using the Supermix reagent purchased from Bio‐Rad (Bio‐Rad laboratories) based on the assay conditions provided by the assay kit manufacturer. Finally, the expression of PTEN mRNA (Forward primer: 5′‐ TGAGTTCCCTCAGCCATTGCCT‐3′; Reverse primer: 5′‐GAGGTTTCCTCTGGTCCTGGTA‐3′) in each sample was determined using the 2^−ΔΔ^ *^Ct^* approach. And beta‐actin (Forward: 5′‐ CATTGCTGACAGGATGCAGAAGG‐3′; Reverse: 5′‐TGCTGGAAGGTGGACAGTGAGG‐3′) was used as the internal reference gene.

### 2.4 RNA pull‐down assay

An RIP assay was performed with an MS2‐binding protein (MS2bp) or GFP antibodies. The plasmid containing full‐length LINC00470 and the MS2bs element was transfected into KCL22 cells with a plasmid expressing MS2bp‐GFP. The RIP assay was then conducted using GFP antibodies, and the LINC00470‐bound mRNA was analysed by using RNA sequencing. The detailed operation of the RIP assay could be found in the directions provided by the assay kit manufacturer on the product manual (Sigma Aldrich).

### 2.5 MeRIP assay

METTL3, WTAP and METTL14 can work as m6A methyltransferases. In this study, we investigated which m6A methyltransferase played a role in m6A modification of PTEN mediated by LINC00470. To achieve this goal, a MeRIP assay was carried out by utilizing METTL3, WTAP and METTL14 antibodies provided in a MeRIP assay kit (Thermo Fisher Scientific) based on the directions provided by the assay kit manufacturer on the product manual.

### 2.6 Vector construction, mutagenesis and luciferase assay

A luciferase reporter assay was carried out in this study to study the regulatory relationship between PTEN promoter and LINC00470. In brief, the wild‐type PTEN promoter containing the LINC00470 binding site was cloned into a pMIR‐REPORT luciferase vector (Ambion). At the same time, site directed mutagenesis was carried out using a Quick Change II mutagenesis kit (Stratagene) based on the directions provided by the assay kit manufacturer on the product manual to induce a single site mutation in the LINC00470 binding site of PTEN promoter, and the mutant PTEN promoter sequence was also cloned into the pMIR‐REPORT luciferase vector to generate mutant PTEN plasmid. In the next step, wild‐type and mutant luciferase vectors were co‐transfected into KCL22 and K562 cells with LINC00470, and the luciferase activity of transfected cells was detected after 48 hours of transfection using a Wallac 1420 luminometer (Perkin Elmer) in conjunction with a Bright Glo luciferase reporter assay kit based on the directions provided by the assay kit manufacturer on the product manual.

### 2.7 Western blot analysis

In this study, Western blot analysis was carried out to determine the expression of PTEN, METTL3, AKT, p‐AKT, LC3II, Beclin‐1, ATG7 and ATG5 proteins in each sample. In brief, total protein in each sample was isolated using a RIPA buffer following the directions provided by the manufacturer on the product manual (Sigma Aldrich). Then, 100 µg of isolated in each sample were separated on a 12% NuPAGE SDS‐PAGE gel (Invitrogen) and then blotted onto a nitrocellulose membrane. After the membrane was blocked at 4°C overnight using a Western Blocking Reagent (Roche) based on the directions provided by the reagent manufacturer on the product manual, the membrane was subsequently probed with anti‐PTEN, anti‐METTL3, anti‐AKT, anti‐p‐AKT, anti‐LC3II, anti‐Beclin‐1, anti‐ATG7 and anti‐ATG5 primary antibodies as well as HRP‐conjugated secondary antibodies following the incubation directions provided by the antibody manufacturer on the product manual (Abcam). Finally, the relative expression of PTEN, METTL3, AKT, p‐AKT, LC3II, Beclin‐1, ATG7 and ATG5 proteins in each sample was visualized and analysed by using an ECL assay kit (Perkin Elmer) in conjunction with an X‐ray apparatus based on the directions provided by the assay kit manufacturer on the product manual.

### 2.8 Statistical analysis

All statistical analysis was carried out by utilizing SPSS 16.0 software (SPSS). Student's *t* test was used to compare the differences between two groups. One‐way ANOVA was used to compare the differences between multiple groups. All results were shown as mean ± SEM *P* < .05 was deemed statistically significant.

## 3 RESULTS

### 3.1 LINC00470 down‐regulated PTEN expression

**FIGURE 1:**
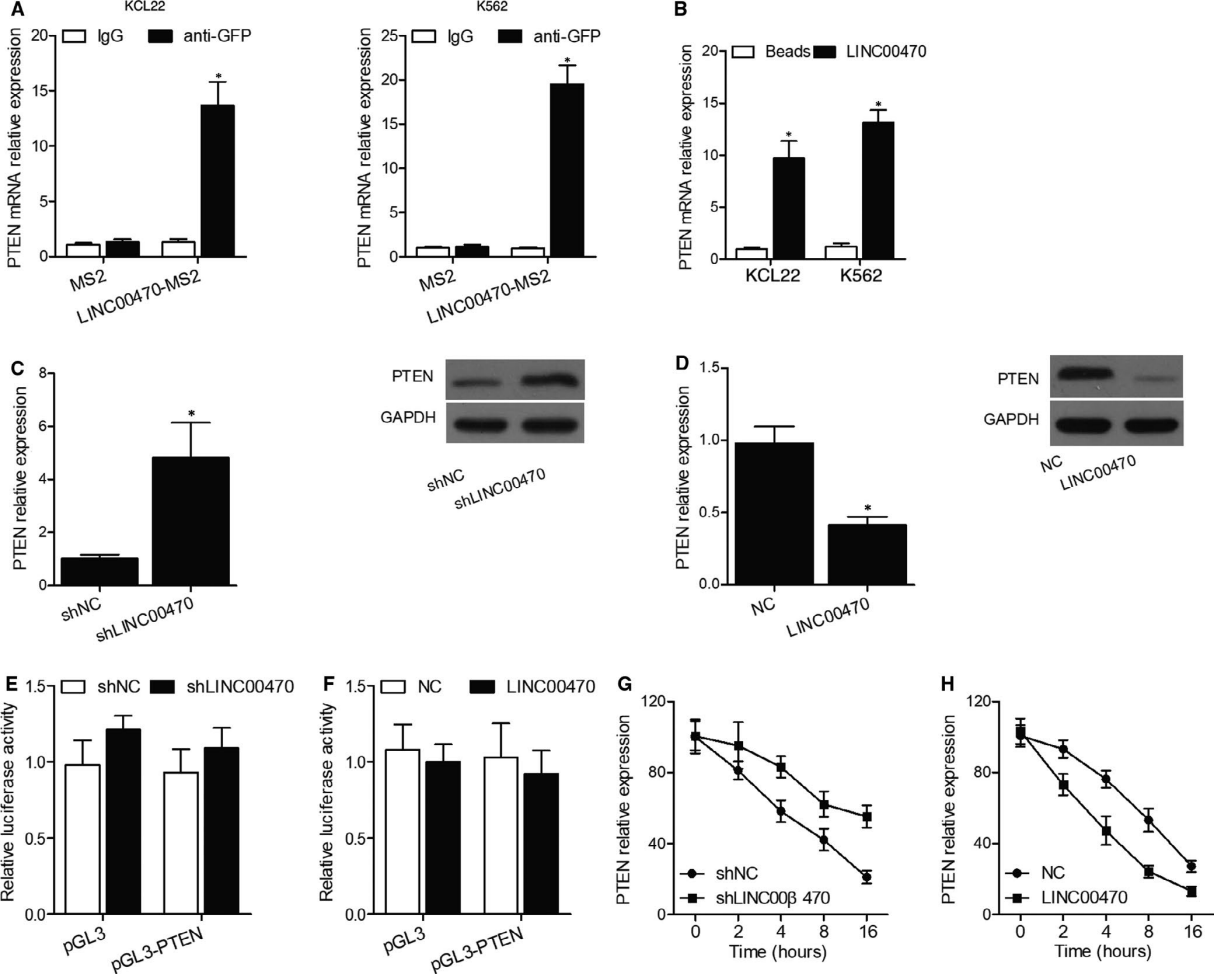
LINC00470 down‐regulated PTEN expression and promoted the degradation of PTEN mRNA. A, LINC00470 in KCL22 and K562 cells was enriched for PTEN mRNA in comparison with the empty vector or IgG (*P value < .05 vs IgG group); B, RNA pull‐down assay of PTEN mRNA expression in KCL22 cells (*P value < .05 vs Beads group); C, Western blot analysis indicated that the knockdown of LINC00470 up‐regulated PTEN expression (*P value < .05 vs shNC group); D, Western blot analysis indicated that the overexpression of LINC00470 down‐regulated PTEN expression (*P value < .05 vs NC group); E, Luciferase reporter assay of PTEN promoter showed that the knockdown of LINC00470 in KCL22 cells had no effect on the luciferase activity of PTEN promoter; F, Luciferase reporter assay of PTEN promoter showed that the overexpression of LINC00470 in KCL22 cells had no effect on the luciferase activity of PTEN promoter; G, The knockdown of LINC00470 extended the half‐life of PTEN mRNA in KCL22 cells; H, The overexpression of LINC00470 shortened the half‐life of PTEN mRNA in K562 cells

An RIP assay was performed with an MS2‐binding protein (MS2bp) or GFP antibodies. The plasmid containing full‐length LINC00470 and the MS2bs element was transfected into KCL22 cells with a plasmid expressing MS2bp‐GFP. As shown in Figure 1A, LINC00470 in KCL22 and K562 cells was significantly enriched for PTEN mRNA in comparison with the empty vector or IgG (Figure 1A), and the RNA pull‐down assay further validated the regulatory relationship between LINC00470 and PTEN mRNA (Figure 1B). Moreover, Western blot analysis of the relative expression of PTEN protein indicated that the knockdown of LINC00470 evidently up‐regulated PTEN expression (Figure 1C), while the overexpression of LINC00470 significantly down‐regulated PTEN expression (Figure 1D).

### 3.2 LINC00470 promoted the degradation of PTEN mRNA

The luciferase reporter assay carried out using the PTEN promoter showed that the knockdown of LINC00470 in KCL22 cells (Figure 1E) or the overexpression of LINC00470 in K562 cells (Figure 1F) had no actual impact on the luciferase activity of PTEN promoter, indicating that LINC00470 regulated the expression of PTEN in a post‐transcriptional manner possibly by affecting the degradation of PTEN mRNA. Therefore, we measured the loss of PTEN mRNA in KCL22 cells treated with α‐amanitin, an inhibitor of RNA synthesis. Accordingly, the knockdown of LINC00470 extended the half‐life of PTEN mRNA in KCL22 cells (Figure 1G). On the other hand, the overexpression of LINC00470 shortened the half‐life of PTEN mRNA in K562 cells (Figure 1H), thus validating that LINC00470 down‐regulated PTEN expression via promoting the degradation of PTEN mRNA.

### 3.3 LINC00470 positively regulated the m6A modification of PTEN mRNA

**FIGURE 2:**
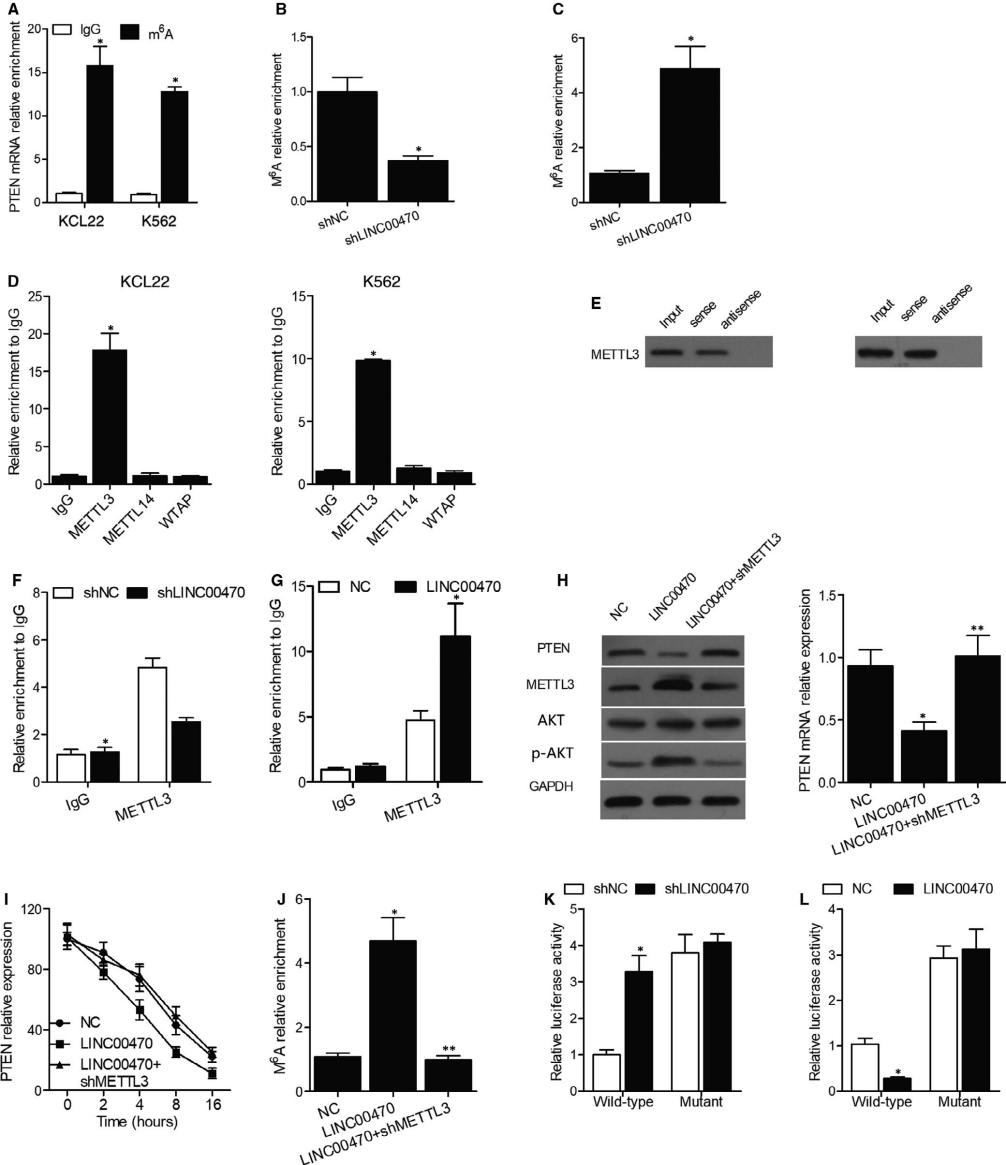
LINC00470 down‐regulated PTEN expression by positively regulating the m6A modification of PTEN mRNA via RNA methyltransferase METTL3. A, The MeRIP assay showed that PTEN mRNA was significantly enriched by m^6^A antibody compared to the negative control IgG in KCL22 and K562 cells (*P value < .05 vs IgG group); B, The relative enrichment of m^6^A was inhibited by the knockdown of LINC00470 in KCL22 cells (*P value < .05 vs. shNC group); C, The relative enrichment of m^6^A was promoted by the overexpression of LINC00470 in K562 cells (*P value < .05 vs shNC group); D, An RIP assay using METTL3, METTL14 and WTAP antibodies showed that METTL3 antibody enriched the transcripts of LINC00470 compared with METTL14 and WTAP antibodies (*P value < .05 vs IgG group); E, RNA pull‐down assay exhibited an obvious interaction between LINC00470 and METTL3; F, The binding of METTL3 to PTEN mRNA was suppressed in LINC00470‐depleted KCL22 cells (*P value < .05 vs shNC group); G, The binding of METTL3 to PTEN mRNA was augmented in LINC00470‐overexpressing K562 cells (*P value < .05 vs NC group); H, The knockdown of METTL3 in K562 cells rescued the down‐regulation and degradation of PTEN mRNA induced by LINC00470 (*P value < .05 vs NC group; **P value < .05 vs LINC00470 group); I, The knockdown of METTL3 in K562 cells rescued the down‐regulation and degradation of PTEN protein induced by LINC00470; J, The knockdown of METTL3 in K562 cells recovered the normal level of PTEN m^6^A modification increased by LINC00470 (*P value < .05 vs NC group; **P value < .05 vs LINC00470 group); K, The knockdown of LINC00470 increased the luciferase activity of wild‐type PTEN in KCL22 cells (*P value < .05 vs shNC group); L, The overexpression of LINC00470 decreased the luciferase activity of wild‐type PTEN in K562 cells (*P value < .05 vs NC group)

As m^6^A modification could affect lncRNA‐mediated stability of RNA, the relevance of m^6^A modification in LINC00470‐mediated regulation of PTEN mRNA degradation was explored. As shown in Figure 2A, the MeRIP assay showed that in KCL22 and K562 cells, PTEN mRNA was significantly enriched by m^6^A antibody compared to the negative control IgG, and the relative enrichment of m^6^A was markedly inhibited by the knockdown of LINC00470 in KCL22 cells (Figure 2B) but evidently promoted by the overexpression of LINC00470 in K562 cells (Figure 2C), indicating the positive regulatory effect of LINC00470 on the m^6^A modification of PTEN mRNA.

### 3.4 LINC00470 down‐regulated PTEN expression via RNA methyltransferase METTL3

To investigate which m^6^A methyltransferase was responsible for LINC00470‐mediated regulation of PTEN mRNA degradation, an RIP assay using METTL3, METTL14 and WTAP antibodies was conducted. As shown in Figure 2D, compared with METTL14 and WTAP antibodies, the METTL3 antibody significantly enriched the transcripts of LINC00470, while the RNA pull‐down assay exhibited the obvious interaction between LINC00470 and METTL3 (Figure 2E). Moreover, the binding of METTL3 to PTEN mRNA was obviously suppressed and enhanced in KCL22 cells depleted of LINC00470 (Figure 2F) and overexpressing LINC00470 (Figure 2G), respectively. Additionally, the knockdown of METTL3 in K562 cells rescued the down‐regulation and degradation of PTEN mRNA (Figure 2H) and protein (Figure 2I) induced by LINC00470, and also recovered the normal level of m^6^A modification in PTEN (Figure 2J). Also, the knockdown of LINC00470 significantly increased the luciferase activity of wild‐type PTEN but not that of mutant PTEN in KCL22 cells (Figure 2K), while the overexpression of LINC00470 decreased the luciferase activity of wild‐type PTEN but not that of mutant PTEN in K562 cells (Figure 2L). Collectively, the above findings suggested that LINC00470 positively regulated the level of m^6^A modification of PTEN mRNA via binding to METTL3.

### 3.5 LINC00470 down‐regulated the expression of LC3II, Beclin‐1, ATG7, and ATG5 via RNA methyltransferase METTL3

**FIGURE 3:**
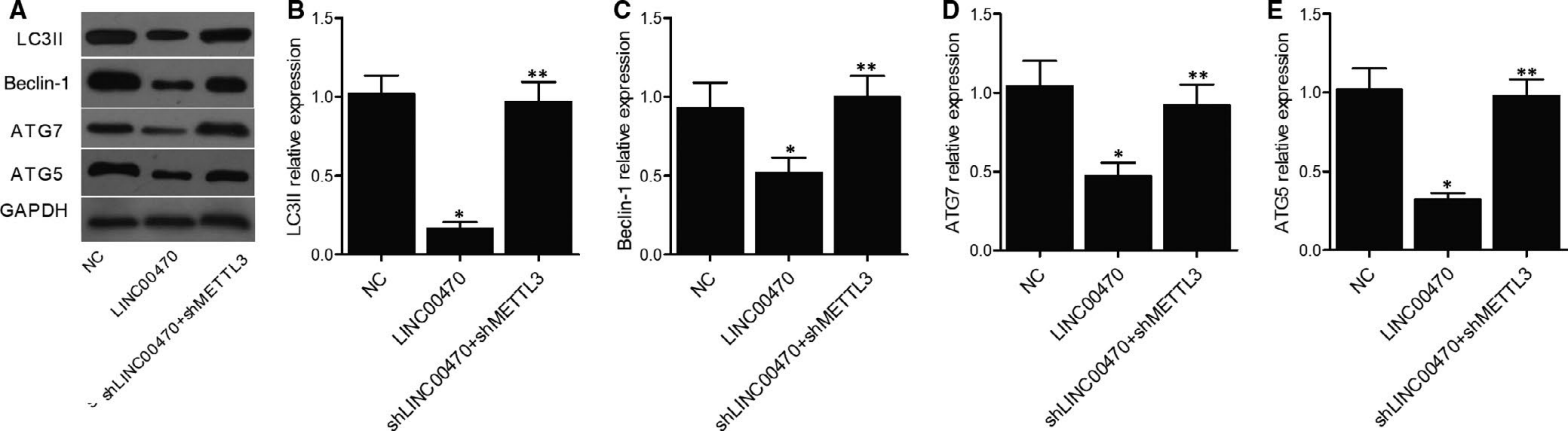
LINC00470 down‐regulated the expression of LC3II, Beclin‐1, ATG7 and ATG5 via RNA methyltransferase METTL3 (*P value < 0.05 vs NC group; **P value < .05 vs LINC00470 group). A, The expression of LC3II, Beclin‐1, ATG7 and ATG5 proteins in different K562 cell groups was measured with Western blot; B, The relative expression of LC3II was decreased in the presence of LINC00470; C, The relative expression of Beclin‐1 was decreased in the presence of LINC00470; D, The relative expression of ATG7 was decreased in the presence of LINC00470; E, The relative expression of ATG5 was decreased in the presence of LINC00470

Moreover, in the K562 cells treated with the negative control, LINC00470 and shLINC00470 + shMETTL3, respectively, the expression of proteins including LC3II, Beclin‐1, ATG7 and ATG5 was measured with Western blot (Figure 3A). As shown in Figure 3, the relative expression of LC3II (Figure 3B), Beclin‐1 (Figure 3C), ATG7 (Figure 3D) and ATG5 (Figure 3E) was all evidently decreased in the presence of LINC00470, while the knockdown of LINC00470 and METTL3 had no significant effect on the expression of these proteins. Therefore, the inhibitory effect of LINC00470 was diminished by the knockdown of METTL3, which was proved to be a necessary factor in the regulation of PTEN expression by LINC00470. In conclusion, it was validated that LINC00470 down‐regulated the expression of LC3II, Beclin‐1, ATG7 and ATG5 via RNA methyltransferase METTL3.

### 3.6 Knockdown of METTL3 recovered the inhibited PTEN expression in chemo‐resistant cells

**FIGURE 4:**
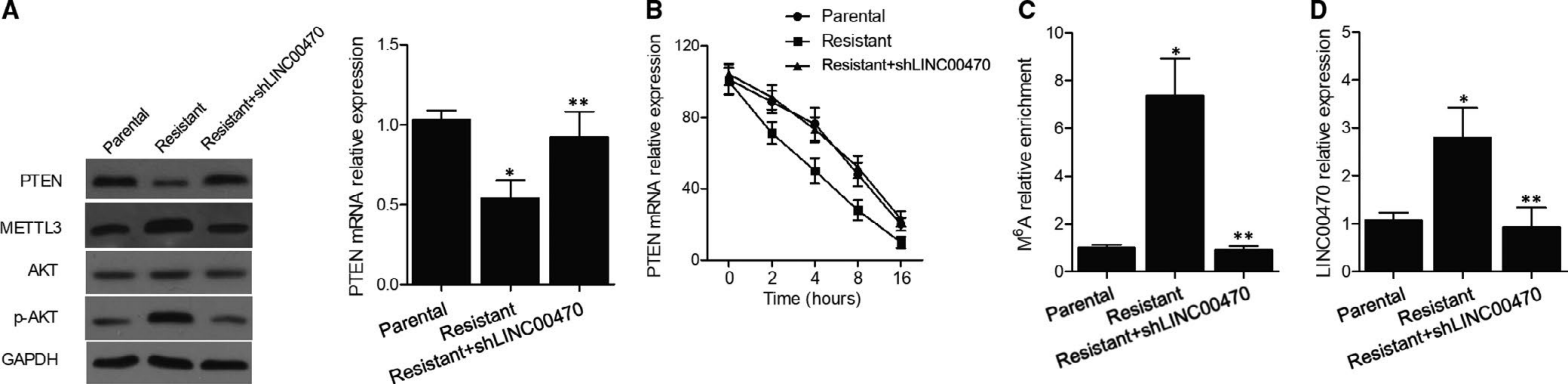
Knockdown of LINC00470 recovered the expression of PTEN inhibited in chemo‐resistant cells (*P value < .05 vs Parental group; **P value < .05 vs Resistant group). A, Western blot analysis showed down‐regulated PTEN protein expression in the chemo‐resistant cell group, and the transfection of shLINC00470 rescued PTEN protein expression; B, PCR showed down‐regulated PTEN mRNA expression in the chemo‐resistant cell group, and the transfection of shLINC00470 rescued PTEN mRNA expression; C, The knockdown of LINC00470 recovered the normal level of PTEN m^6^A modification boosted in the chemo‐resistant cell group; D, The knockdown of LINC00470 recovered the normal level of METTL3 mRNA expression boosted in the chemo‐resistant cell group

**FIGURE 5:**
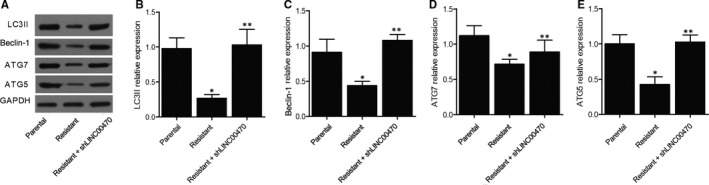
Knockdown of LINC00470 reversed the inhibited expression of LC3II, Beclin‐1, ATG7 and ATG5 proteins in chemo‐resistant cells (*P value < .05 vs Parental group; **P value < .05 vs Resistant group). A, Western blot analysis of LC3II, Beclin‐1, ATG7 and ATG5 protein expression in different cell groups; B, LC3II protein expression was inhibited in chemo‐resistant cells, which were inhibited by the knockdown of LINC00470; C, Beclin‐1 protein expression was inhibited in chemo‐resistant cells, which were inhibited by the knockdown of LINC00470; D, ATG7 protein expression was inhibited in chemo‐resistant cells, which were inhibited by the knockdown of LINC00470; E, ATG5 protein expression was inhibited in chemo‐resistant cells, which were inhibited by the knockdown of LINC00470

Three groups of K562 cells were established as the parental cell group, chemo‐resistant cell group and chemo‐resistant cell + shLINC00470 group, respectively. Western blots and PCR were conducted to observe the PTEN protein (Figure 4A) and mRNA (Figure 4B) expression among different cell groups, and evidently down‐regulated PTEN expression was observed in the chemo‐resistant cell group compared with the parental cell group, while the transfection of shLINC00470 into chemo‐resistant cells rescued PTEN expression. Moreover, the knockdown of LINC00470 also recovered the normal level of m^6^A modification in PTEN (Figure 4C) and relative expression of LINC00470 (Figure 4D) in chemo‐resistant K562 cells. Also, Western blot analysis of LC3II (Figure 5A,B), Beclin‐1 (Figure 5A,C), ATG7 (Figure 5A,D) and ATG5 (Figure 5A,E) in different cell groups all showed evidently down‐regulated expression of above proteins in chemo‐resistant cells, and the expression of above proteins was enhanced by the knockdown of LINC00470.

### 3.7 Knockdown of LINC00470 recovered the normal PTEN expression in ADR mice

**FIGURE 6:**
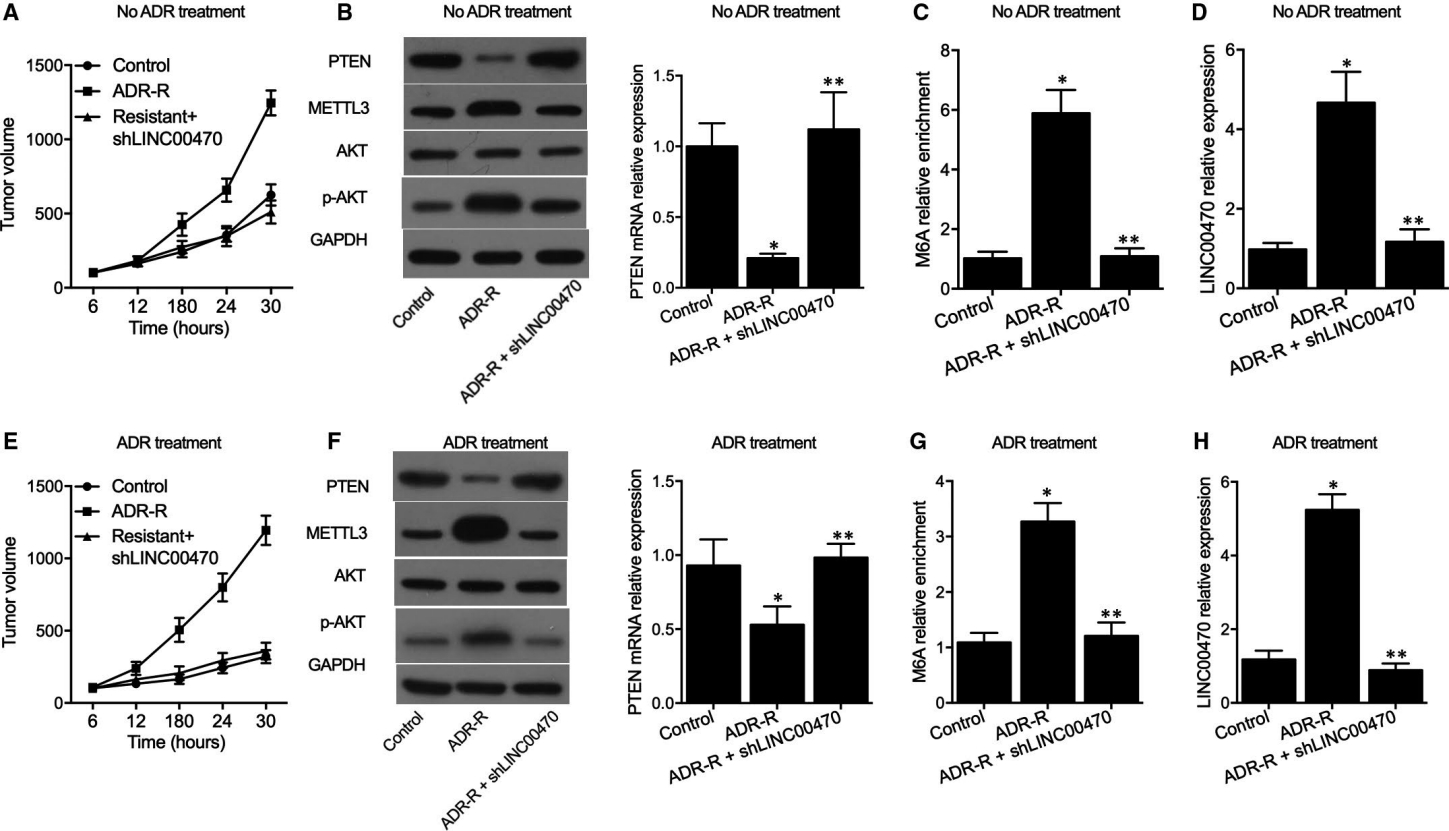
Knockdown of LINC00470 recovered the inhibited PTEN expression in ADR mice (*P value < .05 vs Control group; **P value < .05 vs ADR‐R group). A, Tumour volume was evidently increased in ADR‐R mice, and the presence of shLINC00470 suppressed the tumour growth in ADR mice; B, PTEN mRNA expression was evidently reduced in ADR‐R mice and recovered in ADR‐R + shLINC00470 mice; C, MeRIP assay showed that the knockdown of LINC00470 in vivo could reduce the up‐regulated PTEN m^6^A modification in ADR‐R mice; D, MeRIP assay showed that the knockdown of LINC00470 in vivo could reduce the up‐regulated LINC00470 expression in ADR‐R mice; E, Upon the ADR treatment, tumour volume was evidently increased in ADR‐R mice, and the presence of shLINC00470 suppressed the tumour growth in ADR mice; F, Upon the ADR treatment, PTEN mRNA expression was evidently reduced in ADR‐R mice and recovered in ADR‐R + shLINC00470 mice; G, Upon the ADR treatment, the knockdown of LINC00470 in vivo could reduce the up‐regulated PTEN m^6^A modification in ADR‐R mice; H, Upon the ADR treatment, the knockdown of LINC00470 in vivo could reduce the up‐regulated LINC00470 expression in ADR‐R mice

Three groups of mice were established as the control mice group (transplanted with parental cells), ADR mice group (transplanted with chemo‐resistant cells) and ADR + shLINC00470 group (transplanted with chemo‐resistant cells + shLINC00470), respectively. Tumour volume was evidently increased in ADR mice, and the presence of shLINC00470 suppressed tumour growth in ADR mice (Figure 6A). As shown in Figure 6B, PTEN mRNA and protein expression was evidently reduced in ADR mice but recovered in ADR + shLINC00470 mice. The MeRIP assay showed that the knockdown of LINC00470 in vivo could reduce the levels of PTEN m^6^A modification (Figure 6C) and LINC00470 expression (Figure 6D) markedly up‐regulated in ADR mice. When interfered with ADR treatment, similar results were obtained (Figure 6E‐H).

### 3.8 LINC00470 down‐regulated the expression of LC3II, Beclin‐1, ATG7, and ATG5 via RNA methyltransferase METTL3

**FIGURE 7:**
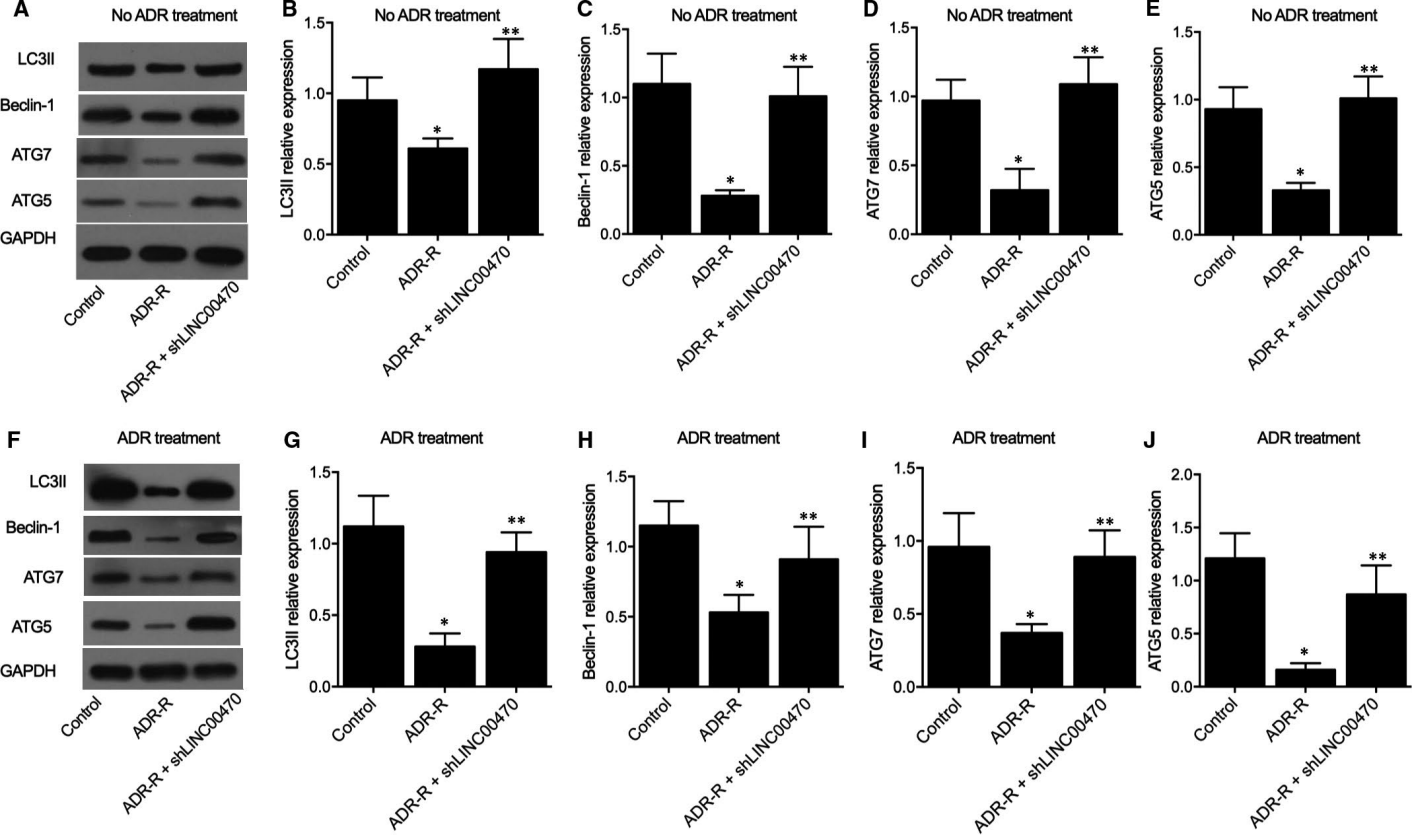
LINC00470 down‐regulated the expression of LC3II, Beclin‐1, ATG7 and ATG5 via RNA methyltransferase METTL3 (*P value < .05 vs Control group; **P value < .05 vs ADR‐R group). A, The expression of LC3II, Beclin‐1, ATG7, and ATG5 proteins in different mouse groups was measured with Western blot; B, The relative expression of LC3II was decreased in ADR‐R mice, while the knockdown of LINC00470 attenuated the relative expression of LC3II induced by ADR; C, The relative expression of Beclin‐1 was decreased in ADR = R mice, while the knockdown of LINC00470 attenuated the relative expression of Beclin‐1 induced by ADR; D, The relative expression of ATG7 was decreased in ADR‐R mice, while the knockdown of LINC00470 attenuated the relative expression of ATG7 induced by ADR; E, The relative expression of ATG5 was decreased in ADR‐R mice, while the knockdown of LINC00470 attenuated the relative expression of ATG5 induced by ADR; F, The expression of LC3II, Beclin‐1, ATG7 and ATG5 proteins in different mouse groups with ADR treatment was measured with Western blot; G, Upon the ADR treatment, the relative expression of LC3II was decreased in ADR‐R mice, while the knockdown of LINC00470 attenuated the relative expression of LC3II induced by ADR; H, Upon the ADR treatment, the relative expression of Beclin‐1 was decreased in ADR‐R mice, while the knockdown of LINC00470 attenuated the relative expression of Beclin‐1 induced by ADR; I, Upon the ADR treatment, the relative expression of ATG7 was decreased in ADR‐R mice, while the knockdown of LINC00470 attenuated the relative expression of ATG7 induced by ADR; J, Upon the ADR treatment, the relative expression of ATG5 was decreased in ADR‐R mice, while the knockdown of LINC00470 attenuated the relative expression of ATG5 induced by ADR

**FIGURE 8:**
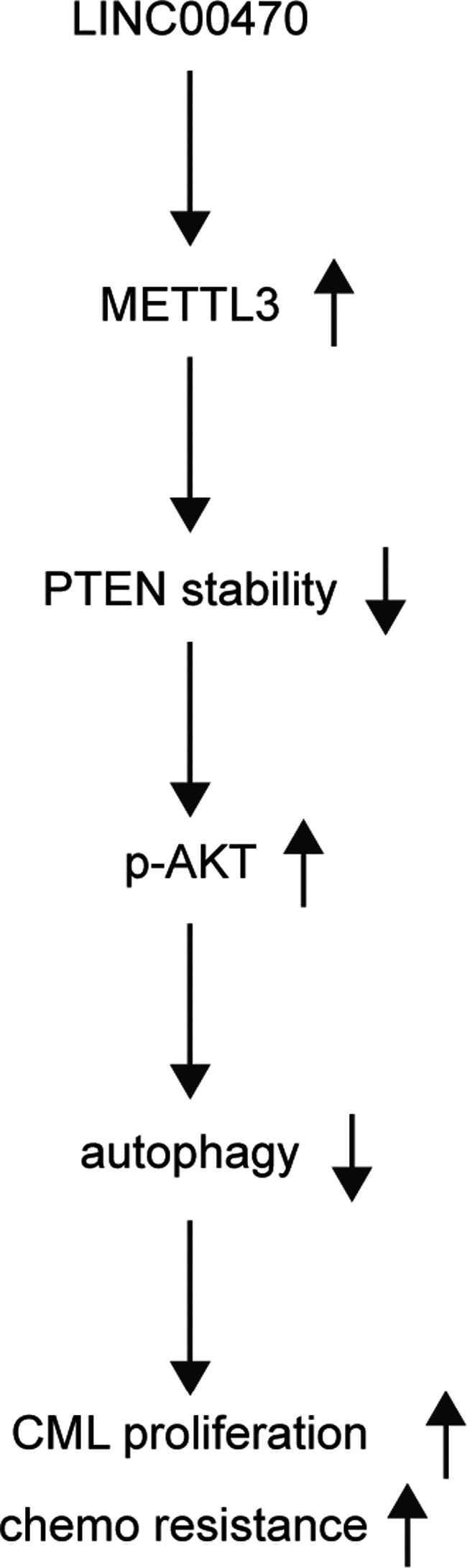
Flow chart of molecular mechanism underlying the effect of LINC00470 on CML

Furthermore, the expression of proteins including LC3II, Beclin‐1, ATG7 and ATG5 was measured in different mouse groups via Western blot (Figure 7A). As shown in Figure 7, the relative expression of LC3II (Figure 7B), Beclin‐1 (Figure 7C), ATG7 (Figure 7D) and ATG5 (Figure 7E) was all significantly decreased in ADR mice, while the knockdown of LINC00470 attenuated ADR‐induced up‐regulation of these proteins. Therefore, according to the in vivo and in vitro results obtained in this study, a molecular mechanism (Figure 8) was established to explain the role of LINC00470 in reducing the stability of PTEN via RNA methyltransferase METTL3, which in turn led to the inhibition of cell autophagy as well as the promotion of chemoresistance in CML. When interfered with ADR treatment, similar results were obtained (Figure 7F,J).

## 4 DISCUSSION

In this study, we found that tumour volume was evidently increased in ADR mice, and the presence of shLINC00470 suppressed tumour growth in ADR mice. The levels of PTEN mRNA and protein were evidently reduced in ADR mice but were recovered in ADR + shLINC00470 mice. The in vivo knockdown of LINC00470 could reduce the levels of PTEN m^6^A modification and LINC00470 expression markedly up‐regulated in ADR mice, along with significantly decreased LC3II, Beclin‐1, ATG7 and ATG5 expression in ADR mice. The LINC00470 lncRNA was recently identified in chromosome 18p11.32. It was demonstrated that LINC00470 lncRNA acts as an oncogene in the pathogenesis of glioblastoma. LINC00470 can also enhance the activity of AKT while inhibiting the ubiquitination of HK1, thereby inhibiting the autophagy of tumour cells while promoting tumorigenesis.^30^ In addition, LINC00470 not only can up‐regulate the expression of ELFN2 via reducing miR‐101 expression, but also regulate the level of ELFN2 methylation by reducing the occupancy of H3K27me3, consequently inhibiting the process of autophagy.^31^ To evaluate the biological role of LINC00471, the link between the aberrant expression of LINC00471 and the expression of hsa‐mir‐150, hsa‐mir‐100, ERMP1, ANP32E, PAPD7, MYO1B, TERF1, PTGIS, as well as VEGFA was studied in patients with a high risk of AML.^32^ Furthermore, it was found that PTEN was down‐regulated in GC via a unique mechanism, while LINC00470 controlled the expression of PTEN by mediating the methylation of m6A induced by METTL3.^10^ In this study, we found that LINC00470 was significantly enriched for PTEN mRNA in comparison with the empty vector or IgG. The knockdown of LINC00470 evidently up‐regulated PTEN expression, while the overexpression of LINC00470 significantly down‐regulated PTEN expression. Although the knockdown or overexpression of LINC00470 had no actual impact on the luciferase activity of PTEN promoter, the knockdown of LINC00470 extended the half‐life of PTEN mRNA while the overexpression of LINC00470 shortened the half‐life of PTEN mRNA, thus validating that LINC00470 down‐regulated PTEN expression via promoting the degradation of PTEN mRNA.

METTL3 acts as a vital member in the complex of m6A methyltransferase. METTL3 was also recently reported to be vital for the progression of hepatocellular cancer, leukaemia, as well as malignant glioma through regulating a diverse assay of downstream target genes.^33^, ^34^, ^35^ More significantly, METTL3 inhibits autophagy through transcription destabilization of TFEB. For that reason, Mettl3 is not able to target the GFP‐TFEB plasmid directly. Nonetheless, it was found that the knockdown of Mettl3 dramatically enhanced the level of GFP‐TFEB proteins by affecting the AMPK‐MTOR signalling.^36^ Vu et al also disclosed that METTL3 may regulate the mRNA level of PTEN in the MOLM‐13 cell line of human myeloid leukaemia.^12^ In addition, it was discovered that the expression of METTL3 was negatively correlated to the expression of PTEN in patients with bladder cancer.^37^ In this study, we found that METTL3 significantly enriched the transcripts of LINC00470, and the binding of METTL3 to PTEN mRNA was obviously suppressed in LINC00470‐depleted cells while the binding of METTL3 in PTEN mRNA was obviously augmented in LINC00470‐overexpressed cells. Additionally, the knockdown of METTL3 rescued the down‐regulation and degradation of PTEN mRNA and protein, as well as the increased PTEN m^6^A modification. Also, the depletion of LINC00470 increased the luciferase activity of wild‐type PTEN, while the overexpression of LINC00470 decreased the luciferase activity of wild‐type PTEN.

PTEN acts as a tumour suppressor in many types of cancer cells to inhibit the process of tumorigenesis as well as the self‐renewal of tumour cells.^38^, ^39^ Inactivated by the HDAC6 inhibitor, PTEN can inhibit the signalling pathways of Notch and PI3K‐AKT‐Mtor in cancer cells to suppress BM1 activity.^40^, ^41^, ^42^ In CML, PTEN was shown to inhibit the self‐renewal as well as survival of LSCs.^22^, ^43^

It was also revealed that PTEN can interact with P62, and HULC can mediate the degradation of PTEN by autophagy. In fact, reduced autophagy enhances PTEN expression, while increased autophagy reduces PTEN expression, suggesting that the degradation of PTEN by autophagy via the interaction of PTEN‐p62 acts as a novel way of tumorigenesis of HULC.^44^ In this study, we found that compared with the negative control, LINC00470 evidently decreased the relative expression of LC3II, Beclin‐1, ATG7 and ATG5 in cells, while co‐transfection of shLINC00470 and shMETTL3 had no significant effect on the expression of these proteins. In addition, we found that chemo‐resistant cells showed evidently down‐regulated PTEN expression compared with the parental cells, and the transfection of shMETTL3 into chemo‐resistant cells rescued PTEN expression. Moreover, the knockdown of METTL3 also recovered the normal level of PTEN m^6^A modification and relative expression of LINC00470, while inhibiting LC3II, Beclin‐1, ATG7 and ATG5 expression in chemo‐resistant cells. Previous study showed that the reduced expression of EPS8 boosted chemosensitivity in K562 cells that were sensitive to imatinib. In addition, Chinni and Sarkar showed that Akt deactivation is a crucial step in the apoptosis of prostate cancer cells induced by I3C.^45^ AKT can promote cell survival and growth by activating downstream targets including XIAP, Bcl‐2, as well as surviving, which were shown to be linked to a poor prognosis YY.^46^, ^47^, ^48^

## 5 CONCLUSION

The findings of this study demonstrated that LINC00470 is a regulator of METTL3, and the deregulation of the LINC00470/METTL3 signalling pathway promoted chemoresistance and suppressed autophagy of CML cells by modulating the stability of PTEN and activing AKT.

## CONFLICT OF INTEREST

None.

## AUTHOR CONTRIBUTION

**Xun Lai:** Conceptualization (equal); Methodology (equal); Project administration (equal); Resources (equal); Writing‐original draft (equal). **Jia Wei:** Conceptualization (equal); Data curation (equal); Software (equal); Validation (equal). **Xue‐zhong Gu:** Data curation (equal); Formal analysis (equal); Software (equal); Validation (equal). **Xiang‐mei Yao:** Conceptualization (equal); Data curation (equal); Formal analysis (equal); Methodology (equal). **Di‐si Zhang:** Investigation (equal); Software (equal); Validation (equal); Writing‐original draft (equal). **Feng Li:** Formal analysis (equal); Methodology (equal); Validation (equal); Writing‐original draft (equal). **Yun‐yan Sun:** Funding acquisition (equal); Methodology (equal); Project administration (equal); Resources (equal); Supervision (equal); Writing‐original draft (equal).

## Data Availability

The data that support the findings of this study are available from the corresponding author upon reasonable request.
